# Supplementary material for: Bacterial Small RNAs in the Genus Herbaspirillum spp
Source: Int J Mol Sci. 2018 Dec 22;20(1):46. doi: 10.3390/ijms20010046 (PMC6337395; doi:10.3390/ijms20010046)
Supplement: Supplementary file 1 [file ijms-20-00046-s001.zip › ijms-392777-final done-supplementary/ijms-392777 -Supplementary Material S1, S2 and S4.docx]

**(Supplementary Material S1)—Species of *Herbaspirillum* with Access Code and Date.**

| **Genus *Herbaspirillum*** | **NCBI Access Code Access Date** | **Access Date** |
| --- | --- | --- |
| *Herbaspirillum seropedicae* SmR1 | NC_014323.1 | 17/02/2016 |
| *Herbaspirillum seropedicae* Z67 | NZ_CP011930.1 | 17/02/2016 |
| *Herbaspirillum lusitanum* P6-12 | NZ_AJHH01000397.1 | 19/03/2016 |
| *Herbaspirillum hiltneri* N3 | NZ_CP011409.1 | 21/03/2016 |
| *Herbaspirillum frisingense* GSF30 | NZ_AEEC02000001.1 | 22/03/2016 |
| *Herbaspirillum*spp. estirpe B65 | NZ_BADI01000775.1 | 22/03/2016 |
| *Herbaspirillum rubrisubalbicans M1* | NZ_CP013737.1 | 22/03/2016 |
| *Herbaspirillum huttiense subsp. putei*  estirpe IAM 15032 | NZ_ANJR01000025.1 | 22/03/2016 |
| *Herbaspirillum autotrophicum* IAM 14942 | NZ_LFLS01000001.1 | 22/03/2016 |
| *Herbaspirillum*spp. estirpe B501 | NZ_BADJ01000320.1 | 22/03/2016 |
| *Herbaspirillum*spp. estirpe GW103 | NZ_AJVC01000003.1 | 22/03/2016 |
| *Herbaspirillum*spp. estirpe RV1423 | NZ_CBXX010000004.1 | 22/03/2016 |
| *Herbaspirillum rhizosphaerae* UMS-37 | NZ_LFLU01000001.1 | 22/03/2016 |
| *Herbaspirillum rubrisubalbicans* spp.  estirpe Os34 | NZ_AMSB01000001.1 | 22/03/2016 |
| *Herbaspirillum rubrisubalbicans* spp.  estirpe Os45 | NZ_AMSA01000003.1 | 22/03/2016 |
| *Herbaspirillum* AU14040 | NZ_CP011930.1 | 22/03/2016 |

**(Supplementary Material S2)—Sequence of ncRNAs Predicted in *H. seropedicae* SmR1 by Infernal 1.1.1**

>5_ureB_sRNA undefined product 5415884:5416169 reverse

5’CTTCCTCGATCTGCTGCGGACAGATGAAGTGGATGTGGCTGTCGATGCCGCCGGCGGTGACGATCATGCCTTCACCCGCGATGATTTCGGTAGCGCCACCGATGGCCATGGTCACGCCAGGCTGGATGTCGGGATTGCCGGCCTTGCCGATGCCGGCGATCTTGCCCGACTTGATGCCGATGTCGGCCTTGACGATGCCCCAGTGATCCACGATCAGGGCATTGGTGATGACGGTATCCATCACGTCCGCATGGGCGCGTTGCGACTGGCCCATGCCGTCGCGGAT3’

>6S undefined product 1383620:1383796 reverse

5’GTACTCCTGCCGTGTTCGCGATTGCCATATATTCCTTGAACCATTTACGAGCATAGGTTGTGGGATCGTTTGATGGGCGTGAGCGTCGCTAGTCCGATGAACCCGAAATTGAACTACTGCAACCAACTTGAACCGTCAGGTTCAGGATGCCGGCACAGCGGCACAGGCGGGGCTTGA3’

>Afu_254 undefined product 4233427:4233498 forward

5’GCTCTTGCTGGCGCTGCTGGACATGTCCGAAGCCGTGGCGCTGGACGCGATGGCGAGATGCTGGGCTTTGC3’

>alpha_tmRNA undefined product 2687581:2687684 forward

5’GCGAACCCTTTGCGGTGAGCAACACCGGCAAGGTCGGCCATCACATGGTGCCCATGCCGCACCTGGGCGTGATCTTGCCGATGCCGCAATGGCAGGCCCTGGCC3’

>AS1726 undefined product 1536374:1536442 reverse

5’GGAAGGGCAACGACAGCACCAGCGCGCAGCCCCAGATGATGGCCTTCTGCACATCCGGGTCCGGGGTCC3’

>ar35 undefined product 3551853:3552014 forward

5’GTACGGCCCGTTCGGCCGAGGGTGAGCTGGTGCTGGTGGTGGGCTTGTCCTTCCTGCTCGATGGCTGTCGCGACATGGAGCGTACGGCAGTGGCGTAAGCTGCGGGCAATATGGGCAAGAAAGCAAAACGGCCGGATCTTCTACGATCCGGCCGTTTTGTTT3’

>ASdes undefined product 788645:788709 forward

5’GATCGAAGTGGCCGCTGACGCGCAGACCCCGATGATCTCGCTGGCCGCTGCCGCGCGCATCGTCG3’

>Bacteria_large_SRP undefined product 4108910:4109005 reverse

5’GGCGGGCCCCTGCGCATTGTGGCATGGTCAACCTGGTCAGGTCGGGAACGAAGCAGCCACAGCCATTTCCCGCAAGTGCCGCAGACAAGGCTCGCC3’

>Bacteria_small_SRP undefined product 4108907:4109005 reverse

5’GGCGGGCCCCTGCGCATTGTGGCATGGTCAACCTGGTCAGGTCGGGAACGAAGCAGCCACAGCCATTTCCCGCAAGTGCCGCAGACAAGGCTCGCCTCC3’

>Betaproteobacteria_toxic_sRNA undefined product 4408979:4409043 reverse

5’TGTCTCCTCCACCCTCCTCAATGGTGTGGATTAAACCCGGAACCACAGTGTCCGGGTTTTTTTTG3’

>beta_tmRNA undefined product 2687527:2687848 forward

5’CCGATACCTGGGTCGATATCGACTTCTTCGGTCATCAGCTCTCGCTGCATCTGGGCGAACCCTTTGCGGTGAGCAACACCGGCAAGGTCGGCCATCACATGGTGCCCATGCCGCACCTGGGCGTGATCTTGCCGATGCCGCAATGGCAGGCCCTGGCCGACCGCCTCAGCGCCAGCGCACAGGTGCAGTTCGTGCTGGCGCCGCAGATCCGCTTCGCCGGCGAACCGGGCGAGCAGGCCACCATGTTCTTTTGCGATCCCTCGGGCAATCCCATCGAGGTCAAGGGCTTTGCCGATCTCCAGCAGGTCTACGCCCGATGAGC3’

>BjrC68 undefined product 2559916:2560015 reverse

5’GCATGCCGCGTTCGTATCAGCTGGATTACGACCAGGGTGGTGAAGTCGAGGGCTATTGGGGAACCTGCGGCGAGACCAGCGTGGCCAACGTGACCTTGAT3’

>Bp1_162 undefined product 4404895:4404959 reverse

5’ACTCGATGGTGCATTACAAGCTGGGTGACAGCGTCCGTGCGCTTGATGCGCATGGCAATGCCTTG3’

>cspA undefined product 1599563:1599935 reverse

5’ATCAGTTACATCTTGTTACTGTTGGCCGATAGAAGGGGGCACGGTTTAAGTGGGGCTGCGTGACGCGCGATGTTTCTTGCTTTAACGGCTCGTGTGATAGTTTATATTTGAAAGATGCTTTTATGGCAACAGGTACTGTCAAGTGGTTCAACGATTCCAAAGGCTTCGGCTTTATCACTCCGGATGACGGCGGCGAAGATCTGTTCGCCCACTTCTCCGCAATCCAGATGAACGGCTTCAAGACCCTGAAAGAAGGTCAAAAAGTCCAGTTTGACGTCACGCAAGGCCCGAAGGGCAAGCAAGCATCGAACATCCAGAACGCCGCCTAAACCCCGGTCTTCTCTGAAAAGCCCCGCCCCGCGGGGCTTTTTTC3’

>crcB undefined product 2800602:2800675 forward

5’ATGTCTCCGATGACCTTCCTGGCGCAACCCTCGATCTGGCTGGATCAGTTGTCTCGTCATCGCGGCACGATTTC3’

>CC3513 undefined product 5090400:5090549 forward

5’CCGCATGCAGATCGCGCTGTCGGAAATGGTGGTCGAGGGCATTTCGACCAACATCCCGCTGCACCGCGAACTGATGGTGGATGCGCGCTTCTTCGAAGGTGGAACCAATATCCATTATCTGGAACATAAGTTGTCGGAACGTCCGGCTTC3’

>CC1840 undefined product 3382967:3383058 forward

5’GAACCGCCGTTGTCGGCCAGACAAGTCTGGCCGACAACGGCGGCGACCGATCAGCGCTTGTCGATCGGCGGCACGTCGCGACGCGGCGAGCC3’

>Chlorobi-RRM undefined product 3830675:3830742 reverse

5’ACCCACTTGCGCGGGGTCGATTGCAGGAAATCCATGCCCTGGGTTTCCTGCTGGTCGTCTTTTTTCTT3’

>Cobalamin undefined product 3038673:3038919 reverse

5’GAACTGGACAAGACCTTCGAGGAACGCGACCACATCAACACCGCCATCGTCAGCGCCATCGACGAGTCGGCCGAGAACTGGGGCGTGAAGGTGCTGCGCTACGAGATCAAGGACCTGACGCCGCCCAAGGAAATCCTGCACGCCATGCAGGCCCAGATTACCGCCGAGCGTGAGAAGCGCGCCCTCATCGCCGCCTCCGAAGGCCGCAAGCAGGAACAGATCAATATCGCCACCGGTGAGCGCGAAG3’

>ctRNA_pGA1 undefined product 3317375:3317443 forward

5’GCTTGGTAATCGACGGATCAAAAAACAATCGCCCGACAGGTTTGCGCCTGTCGGGCGATTTTTTCAGCT3’

>cyano_tmRNA undefined product 3533778:3533837 forward

5’CCAATTCACCCCCACATATCCATGCGGGTCGCTGGTGGAGGTAAGCGGGATCGAACCGCT3’

>drz-agam-1undefined product 1577836:1577922 reverse

5’GGTGTTGACCACCAGGGCGATCTCGTTGTTCTTGACCATGTCCACGATGTGCGGACGGCCTTCCACCACCTTGTTGACGGTGGCCAC3’

>IsrD undefined product 1093595:1093656 forward

5’ATTATTTATACGATTTCCGCTTCGGGAACAGCAAAAAGGCCAGCGCGAAGCTGGCCTTTTCG3’

>IsrG undefined product 3775631:3775718 reverse

5’GGCTGCGGCAACCCAGGCCGCTGCTGCGGCAGCACCCGCACCTGCGGCGGCGTCTGCCTCTACTACGGCAGCGCCTGCTGCTGCAGCC3’

>IsrK undefined product 3533630:3533695 reverse

5’GTGAGTACGGGGGTTCGAATCCCCCAGGGGACGCCAAATAAAAAAGCCCGCGCAAGCGGGCTTTTT3’

>MicC undefined product 2332590:2332667 forward

5’GGCGGCACATCGGCTCACAGGGAGGGAACGCATTCTGCTAGGATCAGTCCTATCAGCTCGTTTCGTCCCGTGCGCCGA3’

>ncr1241 undefined product 3807488:3807558 forward

5’GCAAAGGCTTTCTGATTCTCCCCCGTTTTCCCTTGCAAGAGGCCGGCTGGATGCCGGCCTCTTGCATTTTG3’

>P13 undefined product 3578845:3578907 reverse

5’TGCCGCCGGGGAGGGCGGCGCCTGCCCCAGCTCGGGCGCGACGCGCTGGATCAGCGCCGGCGT3’

>P18 undefined product 4331255:4331365 reverse

5’GTCGACATGACGCGCAGCACGCTCTATGTGCTGCTGCCGCTGTCGGTGGTGTTGGCGCTGGTGCTGGTGCAGCAGGGCAGCATCCAGAACTTCCGCGCCTACCAGGACGTG3’

>P31 undefined product 5496836:5496908 forward

5’AAATTACCGCCGGGAAATCACCTGAAAAGGTGATTTCCGGCGGTAAAGGGATCATCGTCGCCGACGATCCCGA3’

>pfl undefined product 1381288:1381387 forward

5’TTCGTCTCACGTGACTGGCGAAACGCCGGCAAAAGCCGGCCAAGATGGGGATCCATCGGGAAGCGTGAGATTTCAACAGCCGTGCGCCTGGGCAGCCGAA3’

>PyrR undefined product 752778:752894 reverse

5’ATAACCTTTCGGTCGCCCGCACAGCATGCGGGCAAGCGATGCCAACGGTAGCAAACATCCTGCCATCTAGGCTTTACAATAAGAACCTCTTTCGCTTGGCGAAAGAGGTTTTTTTTT3’

>rimP undefined product 2691772:2691837 reverse

AAAGCCCCGGTTTTCCGGGGCTTTTCATTTATCGTAAAGTTTGGAATAATGGCTCTTAACATCCTT

>RNAI undefined product 1330649:1330725 reverse

5’AGAACTTCGCCTGATCTTCAGCATCAGCGACGGCCTTCCCGGCAAGAAAAAACCACGCGCAAGCGTGGTTTTTTTTC3’

>rsmX undefined product 4409813:4409948 reverse

5’ACCTGGGTGCAGCCGAGATGCCGGCGCCGGTCAAGGGCTTGATGAAGGCCATGGCCAAGGTCATGACCACGGTGGCTTACCGCATCTGAGGCAGGGCCTGGCGGGACATGAAAAAAGCGGCCTCGGCCGCTTTTTT3’

>sau-50 undefined product 2940369:2940517 forward

5’TAAATTTCGGAACCTTTGCTGCCTTGATCTTGATCGCTGCACCAGTCTGCGGATTGCGACCGGTACGGGCTGCGCGCTTGCCCACGGCGAAGGTGCCGAAACCTACCAGCGTCACCGTGCCATTCTTCTTCAGCGTGGTCTTTACTGCA3’

>SAH_riboswitch undefined product 4908117:4908206 reverse

5’CGCTGCAAGGAGCGTTGCGACAGGGACATCCCCTGCCAGGCTTGAAGCGGTGAACAGGCGCACGTCGCCTGGCAACCGCGCTCACGTTAC3’

>SpF25_sRNA undefined product 1792781:1792848 reverse

5’TGACAGCGCCGGTATGACGGCGCTGCGAATGTTGAAAAGCCACGAGCGATCCGTGGCTTTTTTTCAT3’

>SpF36_sRNA undefined product 4450248:4450298 forward

5’CAAAAAAGCCTGATCCCGGATGAGATCAGGCTTTTTTATTTCCAAAAAGAA3’

>sR45 undefined product 5447390:5447440 reverse

5’AGCTTTGGCGAGGTCGAGGCCATGAACCGCGACGCCTTCGCCTCGCTGACC3’

>STnc240 undefined product 3961785:3961863 forward

5’ACTGCGTCAAGCAGTAAGCCTCAGCGCTGCCAGTAAAACAAACAGCCGATGCCTGCGAGGGCATCGGCTGTTTGTTTTT3’

>STnc310 STnc310 undefined product 5371582:5371642 forward

5’AGCGAGATGCACCGGCGCAAACGCCGGCCAGGTGGATGCCTGGCCGGCGTTTTTCATGGGC3’

>STnc320 undefined product 5252462:5252517 reverse

5’TTGATGCCTCAACAAGAAAGCGGCGCACCCACCGGGGTGCGCCGCTTTCTTATGTG3’

>STnc350 undefined product 4257834:4257901 reverse

5’TGGCGCTGAAGTGAAGTAGCAAGGAAGGAAGCAAAAGAACGGCCGCATCAGCGGCCGTTCTGTTTTTG3’

>STnc370 undefined product 3751391:3751443 forward

5’AATCTTCGGATTATTTTCGGAAAAAACCGGCCGCAAGGCCGGTTTTTTTTCGT3’

>STnc430 undefined product 245863:245950 reverse

5’AAGAAACTAAAAAACTTAAAAAACTAAAGAGCTAAAAAACCAAGAAAAATAAAAAACGCC

GGCTCGCAAGCCGGCGTTTCCTCATTGC3’

>STnc50 undefined product 3365471:3365564 forward

5’TCTGGGCATCGTAGGCGGCAACCGCGACTGAACCTTGCCTGAACTGCAATAAACCGCAATAAAAAAGCCGGCGCAAGCCGGCTTTTTCACATCG3’

>suhB undefined product 2556514:2556590 reverse

5’AGATTCCCCCGATCTTCGGCGTCTTATATCCCTCCCCTCAGGCCGCCTCCCCCAGGCGGCCATTTTTTTTATCCTTG3’

>sX11 undefined product 2548906:2549016 forward

5’GGGCAGGCCGCGCCGCAGGCTGACCAGTTCCAGCGTGCGGCGCAGGGCGGTGAGCATGCCCAGCGCCAGCACATCGACCTTGAGCAGCTTGAGCGACTCCAGGTCATCCTT3’

>sX4 undefined product 109972:110084 forward

5’TGAGTTCCCTGCACGGAGAGGTGGCCGAGTGGTTAATGGCAGCAGACTGTAAATCTGCCCTCTTACGAGTACGCTGGTTCGAATCCAGCCCTCTCCACCATTAGGCCGGGAGT3’

>sX6 undefined product 1411810:1412066 reverse

5’TCAGCGAAGAAGACGTGATCAAGAAGATCATGCTCAAGGACACCGTCGATGGCGAATTCACCACCACCCAGGACGTGGCCCAAACCGCCGTCTTCCTGGCGGCCTTCCCGACCAATGCTCTGACCGGCCAGTCGGTGGTGGTCAGCCACGGCTGGCATATGCAGTAAGCCGCGCCCGCTGCGACAACCATAGTCGCAACGCGTACAGCCGCCCCGGTGCAGACCGGGGCGGCTTTTTTTGTGCTCTCACCTGCGCTT3’

>symR undefined product 3045259:3045340 reverse

5’CGCCATCCGGTCTGCTGCTGCAGAGGCAGGCCGATGCAGTGATGGCCAATGCGCCCGGGTTCGCGCCCGGGCGCATTGCGCT3’

>TarA undefined product 655476:655577 forward

5’GTGTTGGAAGTCGAAATATTTCCTATTCAATATCGAATAATTTCGATTCGACAATTTTTTTGGGTCTTTTGCCCGAAATTGGCAGGCCTTGGGGCTTGCTTG3’

>Xoo2 undefined product 3686760:3686833 forward

5’ACGTAAGCAATACGTAGGCAACTCGCTGCACAAACAAAAACAGCAGGCCGCATCACCGGGTCTGCTGTTTTTTC3’

>ykkC-III undefined product 4503620:4503684 forward

5’ACATCATGATTCTCCATCATCGCCGCGCGCCGTACCCGGTGTGGCGTGCGCTGGCACGGCGCGGC3’

>yybP-ykoY undefined product 576481:576664 forward

5’AATGCGGCCTTCTCTTTGGGGAGTAGCCAGCTTCCGGATCATCCGGAAGAGCCGTGTCAACATTCTCGGCAGCAGCAGCTGCCGTGGCGCGGGTAGCCAATCGGTAGGCGAGACCATAGACGTTTCCTGCGGCCAGGCTGGGCGCGCAGGCGCGTCCATGGACTTTTCGCCCGGCCAAGGAAT3’

**(Supplementary Material S3)—Cultivation Conditions Employed in the Experiments of RNAseq com H. seropedicae SmR1.**

| **Conditions of Culture** | **Description** |
| --- | --- |
| NFbHPN-Malate (control 1, control 2) | The control condition consists of bacteria grown at 30 ºC in NFbHPN medium, using malate as a carbon source up to D.O 0,8 (TADRA-SFEIR, 2011). |
| NFbHPN-Malate Naringenin (Nar_1, Nar_2) | Bacteria cultured at 30 ºC in NFbHPN medium, using malate as a carbon source in the presence of 100 μM of the flavonoid naringenin, up to D.O. 0.8. (TADRA-SFEIR, 2011). |
| NFbHP-Malate Nitrate (Nitrato_1, Nitrato_2) | Bacteria cultured at 30 ºC in NFbHP medium, using malate as a source of Carbon and 10 mM nitrate as nitrogen source. (BONATO, 2012). |
| Plankton-maize-1day  (PS1_1, PS1_2) | Bacteria recovered from the liquid medium of the hydroponic system (termed seedlings) after one day of corn root inoculation (BALSANELLI, 2013). |
| Planktonic-corn-3 days (PS3_1, PS3_2) | Bacteria recovered from the liquid medium of the hydroponic system (called seedlings) after one day of inoculation in corn root (BALSANELLI, 2013). |
| Adhered-corn- 1 day  (AD1_1, AD1_2) | Bacteria recovered from the root surface of corn (called adhered) after one day of inoculation (BALSANELLI, 2013). |
| Adhered-corn- 3 days  (AD3_1, AD3_2) | Bacteria recovered from the root surface of corn (called adhered) after three days of inoculation (BALSANELLI, 2013). |
| Plankton –Wheat  (PT1, PT2) | Bacteria recovered from the liquid medium of the hydroponic system (called seedlings) after three days of inoculation in wheat root (PANKIEVICZ, 2013). |
| Adhered-wheat  (WR1, WR2) | Bacteria recovered from the root surface of wheat (called adhered) after three days of inoculation (PANKIEVICZ, 2013). |
| NFbHPN-Malate-High Oxygen  (AltoO2_1, AltoO2_2) | Bacteria cultured at 30 °C in NFbHPN medium, using malate as carbon source to D.O. of 0.4. After reaching D.O. 0.4 the bacteria continued to be cultured in this condition for an additional 1.5 hours. (BATISTA, 2013). |
| NFbHPN-Malate-Low Oxygen  (BaixoO2_1, BaixoO2_2) | Bacteria cultured at 30 °C in NFbHPN medium, using malate as carbon source to D.O. of 0.4. At that moment, the bacteria began to be cultivated in microorganism conditions (initial concentration of oxygen 2%) for 1.5 hours. (BATISTA, 2013). |

**References:**

1. Sfeir, M. Z. T. Caracterização funcional de genes de Herbaspirillum Seropedicae regulados pelo flavonóide naringenina. Dissertação (Mestrado em Bioquímica)—Setor de Ciências Biológicas, Universidade Federal do Paraná, Curitiba, **2011**.
2. Bonato, P. Genes do metabolismo de Nitrato em *Herbaspirillum seropedicae*: Regulação transcricional e análise funcional. Dissertação (Mestrado em Bioquímica)—Setor de Ciências Biológicas, Universidade Federal do Paraná, Curitiba, **2012**.
3. Batista, M. B. The Herbaspirillum seropedicae SmR1 Fnr orthologs controls the cytochrome composition of the electron transport chain. [Sci. Rep.](https://www.ncbi.nlm.nih.gov/pubmed/23996052) **2013,** *3*, 2544.
